# Hepatitis B virus, hepatitis C virus and human immunodeficiency virus infections among people who inject drugs in Kuwait: A cross-sectional study

**DOI:** 10.1038/s41598-019-42810-w

**Published:** 2019-04-18

**Authors:** Haya Altawalah, Sahar Essa, Sayeh Ezzikouri, Widad Al-Nakib

**Affiliations:** 1 Department of Microbiology, Faculty of Medicine, Kuwait University, Kuwait City, Kuwait; 2 Virology Unit, Viral Hepatitis Laboratory, Institut Pasteur du Maroc, Casablanca, Morocco

**Keywords:** Infectious-disease diagnostics, Viral infection

## Abstract

Injection drug use (IDU) is one of the most significant risk factors for viral hepatitis (B and C) and human immunodeficiency virus (HIV) infections. This study assessed seroprevalence rates of hepatitis B virus (HBV), hepatitis C virus (HCV) and human immunodeficiency virus (HIV) in people who inject drugs (PWID) in Kuwait. We conducted a cross-sectional study from April to September 2017. A total of 521 consecutive subjects, admitted at Al-Sabah Hospital. The serological and virological markers of HBV, HCV, and HIV were tested  using automated platforms. The mean age of the participants was 32.26 yrs, and the sex ratio (Male/Female) was 15.28. The prevalence rates of HBsAg, anti-HCV, and anti-HIV antibodies were 0.38% (95% CI: 0.07–1.53%), 12.28% (95% CI: 9.65–15.48), and 0.77% (95% CI: 0.25–2.23%), respectively. HCV-RNA was evident in 51.72% (95% CI: 38.34–64.87%) among anti-HCV positive participants. Multivariate analysis showed that the high prevalence of HCV infection amongst PWID is associated with age. Whereas, multivariate analysis revealed no significant associations with age and gender regarding HIV and HBV infections. The results suggest that high rates of HBV, HCV, and HIV infections among injecting drug users than the general population. These findings emphasize the importance of introducing interventions and harm reduction initiatives that have a high impact on reducing needle sharing.

## Introduction

The hepatitis B virus (HBV), hepatitis C virus (HCV) and the human immunodeficiency virus (HIV) are a major global public health issue. Recent reports from the World Health Organization (WHO) have reported that 257 million persons live with chronic HBV infection, 71 million have chronic HCV infection and approximately 36.7 million people living with HIV^1^. People who inject drugs (PWID) are more prone to acquiert blood-borne viruses including HCV, HBV, and HIV through unsafe behaviors such as sharing needles, other injection equipments and drug use paraphernalia^2^. Worldwide, in 2015 an estimated 15.6 million people (10.2–23.7 million) injected drugs of those aged 15–64 years. Among them, 3.2 million (1.6–5.1 million) are women and 12.5 million men (7.5–18.4 million)^2^. Globally, 2.8 million (1.5–4.5 million) PWID are living with HIV, 8.2 million (4.7–12. 4 million) people might be anti-HCV positive and 1.4 million (0.7–2.4 million) people have chronic HBV infection (HBsAg positive)^2^. Furthermore, a recent mathematical modelling analysis shows that several Middle East and North Africa (MENA) countries, PWID contribute dominantly to HIV incidence^3^. To date, no study has reported population-based estimates of HBV, HCV and HIV among PWID in Kuwait. This epidemiological information will add value in creating a national policy for government and policy-makers to allocate appropriate resources and strategically plan for HBV, HCV and HIV prevention interventions. The aim of this study was to determine the prevalence of HBV, HCV and HIV as well as co- and triple-infection rates among PWID in Kuwait.

## Materiel and Methods

### Study population

Between April and September 2017, a cross-sectional study was carried out among injection drug users (IDUs). Inclusion criterias are age more than 17 years, had a history of injecting drug use, be able to provide informed consent and to provide a blood samples for serological and molecular testing. Eligible participants were interviewed confidentially and data were collected. The study protocol was approved by the permanent Committee for Coordination of Medical and Health Research, Ministry of health, Kuwait and the study was conducted in accordance with the ethical guidelines of the 1975 Declaration of Helsinki as reflected in a priori approval by the institution’s human research committee. Written informed consent was obtained from all participants in this study. All methods were performed in accordance with the relevant guidelines and regulations mentioned above.

### Serologic assays

Sera were used for testing HBV, HCV and HIV status. Serum specimens were assessed for antibodies to HIV types 1 and 2 (anti-HIV1/2), HBV surface antigen (HBsAg) and antibody to HCV (anti-HCV) by electrochemical luminescence ECL technology (Cobas 6000 analyser, Roche Diagnostics). Samples showing reactive results were tested using chemiluminescence microparticle immunoassays on the ARCHITECT i2000SR (Abbott Diagnostics, Wiesbaden, Germany).

### Virological testing

Samples showing positive from serology assays results were investigated by molecular assay. Serum HIV-1 and HCV RNA were confirmed using Cobas® TaqMan® 6800 (Roche Diagnostics Deutschland GmbH, Mannheim, Germany) with detection limits of 20 copies/ ml (HIV-1) and 15 IU/ml (HCV).

### Statistical analysis

Qualitative variables were described using percentages and their 95% confidence interval (CI). For categorical variables, the Fisher’s exact test was used. Comparisons of continuous variables between groups were conducted using Student’s t-test. Multivariate logistic regression analyses was used to identify independent risk factors for infections and their respective 95% confidence interval. Statistical procedures were performed with R software. All p-values were two-sided and p < 0.05 was considered statistically significant.

## Results

A total of 521 PWID were screened for HBV, HCV and HIV and during the study period. Of them, 489 (93.9%) were male, and 32 (6.1%) were female. The mean age of the participants in the study was 32.26 years (median 30 years, range 17–82 years) with a significant gender difference (females: mean 28.78 years, range 19–44 years; males: mean 32.48 years, range 17–82 years, p = 0.034) (Table 1).

**Table 1:** Human immunodeficiency virus stratified by gender among PWID in Kuwait.

	All	Male	Female	p-value (male vs. female)
N	521	489	32	
%	100%	93.9%	6.1%	
HIV positive	4	4	0	
HIV positive (%)	0.77	0.82	0	1.000
95% CI	0.25–2.10	0.26–2.23	—	
Median HIV viral load (copies/mL)	49015	49015	—	

95% CI: 95%-confidence interval.

The HIV prevalence in the cohort was 0.77% (95% CI: 0.25–2.23%) (Table 1). The median HIV-RNA viral load was 49015 copies/mL. The differences between males and females were not statistically significant (p = 1.000) (Table 1).

**Figure 1:**
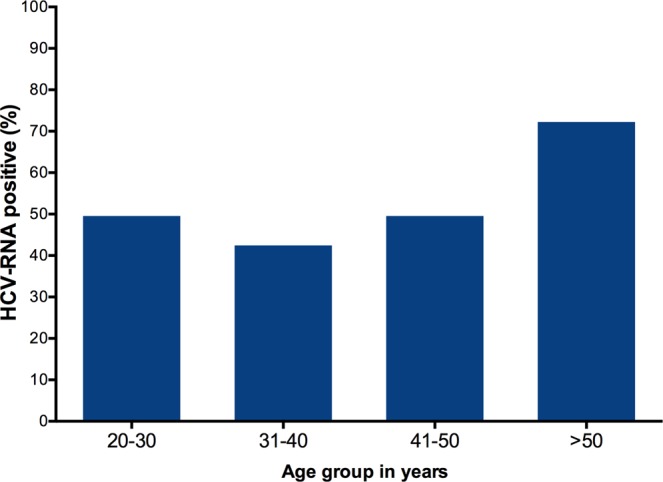
HCV-RNA positivity stratified by age among PWID.

Anti-HCV prevalence was 12.28% (95% CI: 9.65–15.48). The prevalence rates of anti-HCV were 12.88% for males and 3.12% for females (p = 0.159). Of the 64 subjects who were reactive for anti-HCV antibodies, 30 (51.72%, 95% CI: 38.34–64.87%) were HCV-RNA positive while 48.28% had spontaneous HCV-clearance (anti-HCV positive and HCV-RNA negative) (Table 2). In addition, HCV-RNA prevalence correlated with age (Fig. 1).

**Table 2:** Hepatitis C virus infection stratified by gender among PWID in Kuwait.

	All	Male	Female	p-value (male vs. female)
N	521	489	32	
%	100%	93.9%	6.1%	
Anti-HCV-Ab positive	64	63	1	
Anti-HCV-Ab positive (%)	12.28	12.88	3.12	0.159
95% CI	9.65–15.48	10.11–16.25	0.16–18.01	
HCV-RNA (%)	51.72	52.63	0	
95% CI	38.34–64.87	39.09–65.82	—	
Median HCV viral load (IU/mL)	830000	830000	—

The prevalence of HBV (HBsAg positive) was 0.38% (95% CI: 0.07–1.53%) with a non-significant difference between males and females (p = 1.000) (Table 3).

**Table 3:** Hepatitis B virus infection stratified by gender among PWID in Kuwait.

	All	Male	Female	p-value (male vs. female)
N	521	489	32	
%	100%	93.9%	6.1%	
HBsAg positive	2	2	0	
HBsAg (%)	0.38	0.41	—	1.000
95% CI	0.07–1.53	0.07–1.64	—	

HBsAg: Hepatitis B surface antigen, 95% CI: 95% confidence interval.

Only one of the 521 participants (0.19%; 95% CI: 0.01–1.24%) was found to have an HBsAg-positive, anti-HCV positive and HCV RNA negative. HIV/HBV, HIV/HCV co-infection or HIV/HBV/HCV trible infections were not found among IDUs.

Next, we performed multivariate analyses between positive HBV, HCV and HIV status and socio-demographic characteristics (gender and age). As a result, only age showed a significant association with HCV infection; HCV-positive IDUs were significantly older [mean = 42.58 years; standard deviation (SD) = 9.1] than HCV-negative IDUs (mean = 30.81 years; SD = 8.7) (P < 0.0001). In contrast, HCV-positive and HCV-negative IDUs were comparable in terms of gender. Moreover, no demographic factors were associated with HIV or HBV infection (Table 4).

**Table 4:** Independent predictors of infection status in drug users.

	Hepatitis B		Hepatitis C		HIV	
	OR (95% CI)	p-value	OR (95% CI)	p-value	OR (95% CI)	p-value
Age, Y	1.10 (1.01–1.21)	0.290	1.12 (1.09–1.15)	<0.0001	1.01 (0.91–1.11)	0.945
Sex, Male	2.97 (0.14–63.32)	1.000	4.65 (0.62–34.68)	0.160	1.69 (0.09–32.21)	1.000

95% CI: 95% confidence interval.

## Discussion

Drug use remains a major in both developed and developing countries, and is a pivotal factor for a plethora of other social problems^4^. Drug using populations continue to demonstrate a significantly higher prevalence of blood-borne virus infections compared to the general population^5–7^. It is well known that the development of an effective strategy for the control of HBV, HCV, and HIV infections among IDUs is a serious issue for government health authorities. However, there are no data on the rate of blood-borne diseases among PWID in Kuwait. In this cohort, the mean age of the participants (32.26 years) is similar to to previous studies in PWID^8–12^. In addition, most PWID are males (93.9%) and this data corroborate with previous reports highlighting that the prevalence of IDU among men was far higher than in women^2,12–14^.

It was conspicuous that HIV prevalence among IDUs was remarkably high than national infectious level^2^. In this study, we found that the HIV prevalence is 0.77%, which is relative low compared to the prevalence observed among IDUs in other parts of the world. HIV prevalence among PWID varied substantially across geographical regions, from 1.1% in Australasia, 3.6% in the MENA, and 4.5% in western Europe, to 24.7% in eastern Europe and 35.7% in Latin America^2^. In contrast, earlier study and according to the World Bank collection of development indicators, compiled from officially recognized sources, the prevalence of HIV in general population Kuwait was reported at 0–0.1%^15,16^.

The prevalence of HCV among PWID studied reached 12.28%, which was low compared to the rate found among IDUs in other geographical regions. For example, in sub-Saharan Africa, 21.8% of PWID had anti-HCV, 38.6% in the South Asia, 48.1% in the MENA region^2^ and 34.5% in Iran^17^. The lower prevalence relative to other countries is probalbly linked the low levels of sharing of needles, syringes and other drug injection equipment. Moreover, previous studies have reported that the prevalence rate of HCV is up to 10 fold higher than that of HIV^18–20^. In the same line, previous studies carried out in the general popualion showed a prevalence ranging from 0 to 0.8%^21,22^. In contrast, high prevalence was observed among hemodialysis patients (27 to 71%)^23^, thalassemic patients (33%)^24^ and 3% in Kuwaiti individuals with type 2 diabetes^21^. Viremic HCV infections in Kuwaiti PWID were found to 51.72%. This rate is consistent with HCV viremia prevalence among IDUs reported in other countries^8,25^. We found that the factor significantly associated with HCV was older age. This result seems to be in line with pervious study^8^. For HBV and HIV infections, there were no significant associatons with age and gender.

In this study, we found a low HBV prevalence among IDUs (0.38%). In a recent report the highest estimated HBsAg prevalence among PWID was in Thailand (30.5%), China (23.4%) and Myanmar (17.1%). Although countries with the highest prevalence also included the Czech Republic (15.1%), Egypt (13.5%), Belarus (11.2%), Lithuania (10.5%), the Côte d’Ivoire (10.5%) and Azerbaijan (10.4%). Furthermore, the lowest HBsAg prevalence among PWID was in Ireland (0%), Montenegro (0%), Seychelles (0.3%), Bosnia and Herzegovina (0.5%), Syria (0.5%), Maldives (0.5%), Germany (0.7%) and France (0.8%)^2^. Intersetingly, the lower prevalence of HBsAg among PWID in Kuwait could be explained in part by HBV immunity prior injecting drugs. HBV vaccine had been introduced in the national immunization program from 1990 in Kuwait. Interstingly, 37% (195/521) of PWID in our sample were born since 1990 are likely to have been immunized and thus protected against HBV infection. The prevalence rate of HBV carriers is estimated less than 1%, in the general Kuwait population^22,26^.

In conclusion, our results showed that there is a high prevalence of HCV infection, but a low prevalence of HIV, HBV and co-infections among PWID in Kuwait. Moreover, the rate of HBV, HCV, and HIV observed among IDUs in Kuwait is very low compared to other parts of the world. However, these rates are higher than observed among the general poupultion thus underscoring the critical need to improve harm-reduction measures. In addition, effective prevention, public education and comprehensive screening programs with a specific focus on high-risk population should be considered as the top priorities of any health policy decision to reduce blood-borne infections. Finally, we are confident that our work significant impact on future efforts on the HBV, HCV, and HIV transmission among IDUs in Kuwait. Whereas, we are fully aware that the limitation of the current study is the lack of some epidemiological data. Other surveys warrant the enrolment of a larger cohort in the future including other risk behaviors related to unsafe sex, frequency of injecting drug use, needles/syringes sharing, duration of injecting drug use and the number of sexual partner.
